# Correlations of Neck/Shoulder Perfusion Characteristics and Pain Symptoms of the Female Office Workers with Sedentary Lifestyle

**DOI:** 10.1371/journal.pone.0169318

**Published:** 2017-01-06

**Authors:** Jian-Guo Bau, Taipau Chia, Shan-Hua Wei, Yung-Hui Li, Fun-Chie Kuo

**Affiliations:** 1 Department of Biomedical Engineering, Hungkuang University, Taichung, Taiwan, ROC; 2 Department of Safety, Health and Environmental Engineering, Hungkuang University, Taichung, Taiwan, ROC; 3 Neurology Surgery, Cheng Ching General Hospital, Taichung, Taiwan, ROC; 4 Department of Computer Science and Information Engineering, National Central University, Taoyuan, Taiwan, ROC; NETHERLANDS

## Abstract

**Aim:**

Modern office workers are often impacted by chronic neck/shoulder pain. Most of the previous studies which investigated the relationship of the occupational factors and musculoskeletal symptoms had adopted questionnaire survey. In this study the microcirculatory characteristics and perceived symptoms in neck/shoulder region were compared among office workers with sedentary lifestyle.

**Methods:**

Thirty-seven female office workers were recruited in this study. Microcirculatory flow in neck/shoulder region characterized by the mean blood flow (MMBF value), pulsatile blood flow (PMBF value), and the PMBF/MMBF ratio (perfusion pulsatility, PP) were investigated using Laser Doppler Flowmetry (LDF). A Chinese version of the Standardized Nordic Musculoskeletal Questionnaire (NMQ) were also administered to collect the information of perceived neck/shoulder symptoms. Correlations between the perfusion characteristics and the individual/occupational factors were analyzed using the Spearman test. The difference of the MMBF values between the low-pain group (pain level≤2) and the high-pain group (pain level>2) were compared using the Mann-Whitney U test.

**Results:**

There were 81% participants reported neck or shoulder pain symptoms. The duration of shoulder pain was significantly correlated with the workers’ age and the duration of employment (p<0.01) (n = 37). While both the MMBF and PMBF values in shoulder region were significantly reduced with the workers’ age and the duration of employment (p<0.05) (n = 27). And there was a 54% reduction in the MMBF value of the workers from age of 23 to 47. And the MMBF value of the high-pain group (n = 15) was significantly lower than the value of the low-pain group (n = 15) (p<0.05). The duration of shoulder pain showed a moderately negative correlation with PMBF values (n = 19). Besides, the PP value was moderately correlated with shoulder pain level attributed by the rapid reduction of MMBF values (p = 0.07).

**Conclusion:**

In this study, the LDF method was used for the first time in the workplace in Taiwan. It was demonstrated that the MMBF in shoulder region were affected by aging effect and towards lower value at higher pain level. Impaired microcirculation caused by age effect, when coupled with sedentary lifestyle, was found to be more likely to evoke ischemia shoulder pain. Further studies are needed to assess current indicator, PP value, and the underlying mechanism of pain caused by sedentary lifestyle.

## Introduction

Since the early 1980s, the use of video display unit was dramatically increased[1]. Today, extensive use of laptop and cellphones with touch panels increases the risk of physical exposure. In 1990, the risk of physical inactivity was not categorized as a health risk factor. Now it is one of the top ten health risk factors[2]. Sedentary workforce is now facing an urgent priority in addressing prevention of musculoskeletal disorders (MSD) or metabolic diseases.

An early study had shown that the health hazards of office workers included neck, shoulder and low back pain, etc[3]. A study of 124 symptomatic female office workers had demonstrated the association between job stress, workstyle, upper extremity pain and function[4]. Prolonged sitting is prevalent in the workplace and is associated with adverse health markers[5]. In a recent survey, the incidence rate of computer users for neck, shoulder and arm/hand symptoms was 67, 41 and 47 cases per 100 person, respectively[6]. Many workers with chronic nonspecific musculoskeletal pain who continued to work reported poor to moderate work ability and work performance[7].

At the end of last decade, there were many studies involving the intervention program for office workers with neck/shoulder discomforts, the most common outcome measurements were self-reported neck/shoulder symptoms[8–11]. To date, more quantitative outcome measures were applied including measurements of systolic blood pressure, body fat percentage and maximal oxygen uptake[9]; using surface electromyography to monitor muscle activity[12]; using Near-Infrared Spectroscopy to measure trapezius muscle oxygenation[13]; or using a digital dynamometer to measure the back muscle strength[14]. Poor microcirculation may also increase the risk of neck/shoulder discomfort, but few outcome measures of blood perfusion in microvascular flow were investigated.

Recent studies showed that Laser Doppler Flowmetry (LDF) is a valuable and reliable method for diagnostics of microcirculation and perfusion of post-operation or clinical conditions, for assessment of autoregulation and the effect of treatment, the total mean blood flow of the arm or for experimental studies and research[15–19]. Strom’s study had showed that pain in the active side correlated positively with blood flux in the pain-afflicted subjects during 90 min of office-work[20]. Our previous study had shown that there was a significant difference in mean microcirculatory blood flow (MMBF) values between student groups with different calf flexibility[21]. However, LDF was rarely used in the assessment of microcirculatory health in office workers.

The objective of this study is to investigate the relationship between the characteristics of microvascular flow and the perceived symptoms in neck/shoulder region of the office workers with sedentary lifestyle using a non-invasive LDF method. Convenience sampling was used and only female participants joined this study. Novel signal processing of perfusion signal within one heart beat was adopted to evaluate the characteristics of microvascular flow. By applying the LDF methodology, this paper should be considered as explorative.

## Methods

### Participants

Total of 37 female, full-time office workers with sedentary lifestyle (SOW), were informed and joined this program with written consent. They were recruited from two hospitals, one factory and one university. All the SOW in the factory (n = 5) have no perceived shoulder pain symptoms (pain level = 0). The SOW (n = 32) in the hospitals and the university performed similar administrative work, such as counter service and operators, with shorter break time and relatively high job demands, five of the workers reported no perceived shoulder pain symptoms. All the SOW (n = 37) spent less than one hour per week on physical activity. Potential participants with a history of accidents, traumatic injuries or surgical treatment in the neck or upper limb regions were excluded. This study was approved by the ethics committee of Kuang Tien General Hospital, Taiwan, with approval number of 10132.

### Outcome Measurements

In order to investigate the relationship between the perfusion characteristics of microvascular flow and the perceived pain symptoms in neck/shoulder region, the LDF technology and questionnaires were applied in this study. The information of personal and occupational characteristics such as age, body mass index (BMI), general physiological parameters (diastolic blood pressure, systolic blood pressure and heart rate), duration of employment, working time and exercise habit were collected in the questionnaires administered. Thirty SOW agreed to attend the perfusion measurement during the break time of the workday. Seven workers failed to complete the LDF measurement only because of tight schedule. The microcirculatory signals were then processed and the perfusion characteristics were obtained. The LDF measurements were performed by well-trained researchers[21].

#### Questionnaire

A Chinese version of Nordic Questionnaire of musculoskeletal symptoms questionnaire (NMQ) modified by the Taiwan Institute of Occupational Safety and Health was used to collect the data of perceived musculoskeletal symptoms of the participants (n = 37)[22]. The information of perceived symptoms was compared to the subjective LDF measurements for each participant. Three major factors of perceived symptoms considered in this study included the level of neck/shoulder pain intensity, the duration of shoulder pain and the effects of shoulder pain symptom. The level of neck/shoulder pain intensity was rated on a 10-level visual analog scale indicated as 0 (no pain) to 10 (unbearable pain). The duration of shoulder pain (e.g. what is the total length of time that you had shoulder pain) was divided into a 6-level scale: 1 (one month), 2 (three months), 3 (six months), 4 (one year), 5 (three years) and 6 (more than three years). The effects of shoulder pain symptom were divided into a 6-level scale: 1 (no limit on leisure activity or work activity), 2 (mild limit on work activity), 3 (quite a bit limit on my work activity), 4 (been absent from work due to pain symptom), 5 (significant limit on my leisure activity), 6 (shoulder pain has prevent you from doing your normal work most of the time).

#### Laser Doppler Flowmetry Measurement

In our previous study, different microcirculatory characteristics were observed in relation to calf flexibility[21]. Herein, the LDF measurement was further applied to obtain the perfusion characteristics of the participants with shoulder pain symptoms in addition to the information obtained from questionnaires. The participants were asked to stay in the room with temperature maintained at 25–26°C for at least 20 minutes and then a duration of 10-mimutes LDF signal was measured in neck/shoulder region (45% of the distance from C7 to acromion) while resting in a supine position. Variables were controlled including no consumption of coffee or alcoholic-containing drinks, no staying up late, and no exercise on the trail day. The output laser power of the applied LDF system (VMS-LDF1-HP, Moor Instruments, UK) was less than 20 mW (Class 3R per IEC 60825–1:2007) with a wavelength of 785 nm, and a non-invasive skin probe (VP1-V2-HP). The depth of the LDF measurement is down to approximately 1.6 mm[23]. The electrocardiograph (ECG, EBI100C, BIOPAC system, U.S.A.) signals were sampled with the LDF perfusion signal synchronously via an analog-to-digital converter (ADLINK, PCI-9111DG, Taiwan) with sampling rate of 1024 Hz following the safety requirements. The detected LDF signals were processed with the modified beat-to-beat algorithm, from which the average LDF waveform of one heartbeat was obtained. Three parameters of the perfusion characteristics were then defined. From the signal processing, the PMBF value represents the mean intensity of pulsatile microcirculatory blood flow and the MMBF value represents the mean microcirculatory blood flow. Both of them were expressed in an arbitrary unit. The perfusion pulsatility (PP) was defined as the ratio of PMBF to MMBF (PMBF/MMBF). The detailed signal processing of PMBF, MMBF and PP values was described in our previous study[21].

As the aim of this study is mainly to evaluate the perfusion characteristics and pain symptoms of the SOW, so the shoulder side with pain symptom or the side not frequently used were regions of interested. The measured region was postulated to have poor microcirculatory perfusion.

#### Statistics

Statistical analysis was performed using SPSS version 14.0 (SPSS, Inc., Chicago, IL, USA). In this study, the correlations of the perceived neck/shoulder symptoms and the PMBF/MMBF/PP values of the participants were analyzed using the Spearman’s test. When the value of the Spearman correlation coefficient lies around ± 1, it reflects strong association between the two variables. The difference of the MMBF values between the two pain groups was compared using the Mann-Whitney U test, as they are two independent groups with limited samples. A two-tailed significance level of 5% was adopted.

## Results

According to the questionnaire survey, there was ~81% of the SOW had no regular exercise habit; the other 19% workers only exercised regularly about one hour per week. The personal and occupational characteristics are shown in Table 1(S1 Data). In BMI measurement, there were seven subjects were underweight (BMI <18.5), nine subjects were overweight (BMI = 25~30), three subjects were obese (BMI >30). In personal characteristics, the mean heart rate and blood pressure were in normal range. The average duration of employment of the workers was more than 6 years. There were also 30 participants (81%) of the SOW reported neck or shoulder pain symptoms (level of pain>1).

**Table 1:** Characteristics of the office workers with sedentary lifestyle (n = 37).

Personal characteristics	Mean±SD	Occupational characteristics	Mean±SD	NMQ survey	Mean±SD
**Age (years)**	32.2±6.5	**Duration of employment (months)**	72.8±61.4	**Neck pain level**	3.0±2.3
**BMI**	22.6±4.5	**Working time/week (hours)**	43.8±11.1	**Shoulder pain level**	2.6±2.4
**Heart rate (min**^**-1**^**)**	71.2±8.2	**Break time/day (mins)**	33.4±33.6	**Duration of shoulder pain**	2.3±2.4
**DBP/ SBP (mmHg)**	71.4±12.0/ 112.8±14.8			**Effect of shoulder pain**	1.2±1.2

Table 2 exhibits the correlations among neck and shoulder pain symptoms of all office workers (n = 37). It was shown that the level of neck pain was significantly correlated with the level of shoulder pain, shoulder pain duration and the effect of shoulder symptom. Among all participants, thirty workers agreed to complete the LDF measurements. Table 3 showed the correlations of the two measurements (the NMQ symptoms scales and the LDF perfusion scales) and the personal/occupational characteristics. The results showed that the duration of shoulder pain was significantly correlated with the workers’ age and the duration of employment (p<0.01) (n = 37). The pain symptoms were significantly and negatively correlated with the break time during the workday (p<0.05) (n = 37). The PMBF and MMBF values were significantly and negatively correlated with the workers’ age and the duration of employment (p<0.05) (n = 27).

**Table 2:** Correlations between the perceived shoulder and neck symptoms in NMQ survey of the office workers (n = 37)^a^.

	Level of pain	Pain duration (shoulder)	Symptom effect
**Level of pain (neck)**	0.737**		
**Level of pain (shoulder)**	1		
**Pain duration (months) (shoulder)**	0.733**	1	
**Symptom effect (shoulder)**	0.860**	0.671**	1

*: p <0.05

**: p<0.01

^a^: analyzed by spearman test.

**Table 3:** Correlations between the personal/occupational factors, the perceived shoulder symptoms (NMQ^θ^) and the perfusion characteristics (LDF^◊^)^a^.

	NMQ^θ^ Level of pain	NMQ^θ^ Pain duration	NMQ^θ^ Symptom effect	LDF^◊^ PMBF value	LDF^◊^ MMBF value	LDF^◊^ PP value
**Age**	0.282	0.512**	0.260	-0.511*	-0.621*	0.136
**BMI**	0.179	0.290	0.188	-0.391*	-0.242	0.200
**Duration of employment**	0.264	0.437**	0.261	-0.460*	-0.507*	0.282
**Working time/ week**	0.152	0.178	0.158	-0.030	-0.013	-0.008
**Break time/ day**	-0.389*	-0.358*	-0.399*	0.175	0.257	0.236

*: p <0.05

**: p<0.01

^a^: analyzed by spearman test.

^θ^: n = 37 for questionnaire survey.

^◊^: n = 27 for LDF measurement, MMBF>100 were excluded (n = 3).

**Fig 1:**
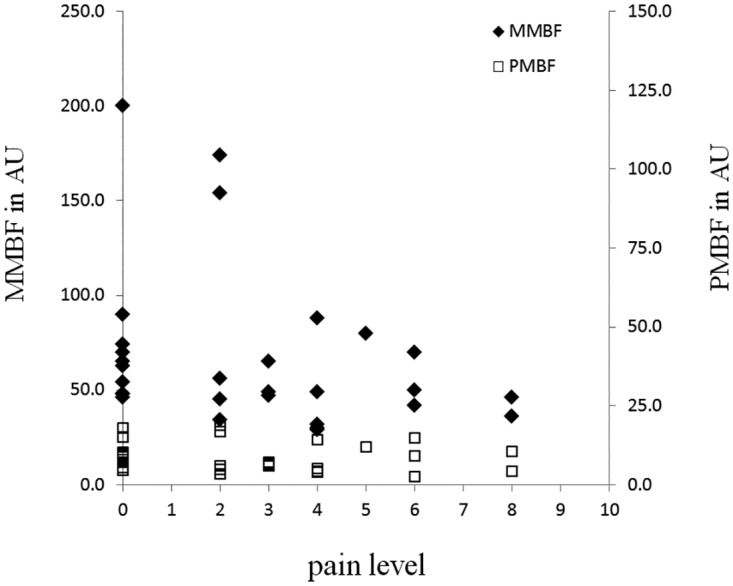
Relationship between the PMBF/MMBF values and the shoulder pain level of the office workers with sedentary lifestyle. (n = 30).

**Fig 2:**
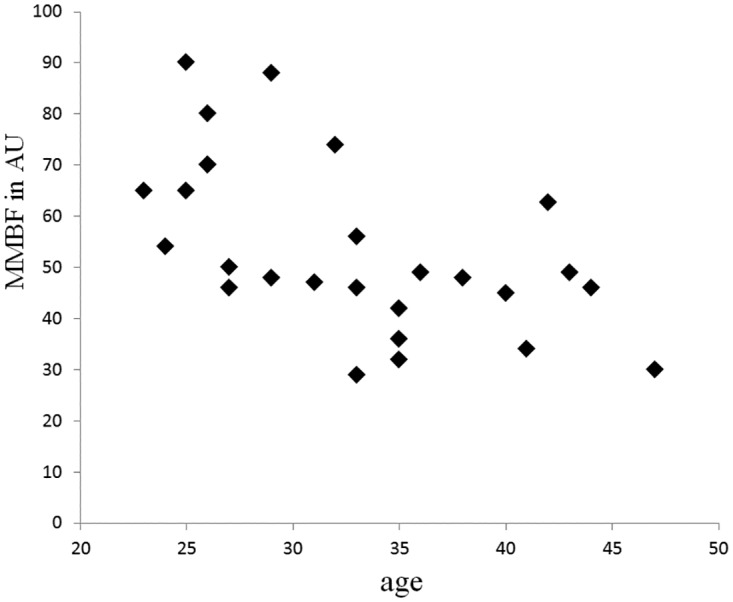
Relationship between the MMBF values and age of the office workers. (n = 27, workers with MMBF > 100 were excluded.).

In comparison of the NMQ scales and the perfusion scales of the participants with shoulder pain level > 1 (Table 4, n = 19). The result showed that the PMBF value was moderately and negatively correlated with the duration of shoulder pain. Interestingly, the ratio of PMBF to the MMBF (PP value) was moderately correlated with shoulder pain level (p = 0.07). In order to further understand the pain symptoms and the microcirculatory characteristics of the workers, Fig 1 depicted the relationship between the PMBF and MMBF values and the level of shoulder pain (n = 30). MMBF values reduced faster than of the PMBF as the level of pain became higher. PMBF values almost maintained at low values at different levels of pain. When the SOW (n = 30) were divided into two pain groups, low-pain group (n = 15) and high-pain group (n = 15). It was found that the MMBF values of the high-pain group were significantly lower than the values of the low-pain group (p = 0.049). Fig 2 demonstrates the relationship between the MMBF values and the age of the office workers (MMBF > 100 were excluded), the MMBF declined with age and there was a 54% reduction from age of 23 to 47. The result of the linear regression analysis showed that there was a decrement of 1.7 arbitrary unit (AU) per year.

**Table 4:** Correlations of the perceived symptoms and perfusion characteristics in shoulder region. ^a^^Φ^(n = 19).

	LDF PMBF value	LDF MMBF value	LDF PP value (PMBF/MMBF)
**NMQ level of pain**	-0.061	-0.218	0.425 (p = 0.07)
**NMQ pain duration (months)**	-0.316	-0.121	-0.201
**NMQ symptom effect**	0.196	0.173	-0.073

*: p <0.05 **: p<0.01

^a^: analyzed by spearman test.

^Φ^: only workers with level of pain ≥ 1 (n = 19) were analyzed.

## Discussion

In addition to the widely used questionnaires, the present results reveal that the microcirculatory characteristics monitored by the non-invasive LDF technique could be a complementary and objective tool for the assessment of the neck/shoulder health. The degradation of microcirculation related to chronic pain and the pain level could be observed by the microcirculatory parameters of the LDF blood perfusion signals. The PMBF and MMBF values were significantly and negatively correlated with the workers’ age and the duration of employment (p<0.05). The MMBF value of the high-pain group is significantly lower than the value of the low-pain group (p<0.05). The PP value (= PMBF/MMBF) was moderately correlated with shoulder pain level attributed by the rapid reduction of MMBF values (p = 0.07).

### Characteristics of the Office Workers

Although the general physiological parameters of most SOW were within the normal range, there were 30 office workers (81%) reported neck or shoulder pain symptoms (mean of shoulder pain level = 2.6±2.4) (Table 1). The significant correlations among the level of pain, the pain duration and the symptom effect revealed that the musculoskeletal disorders prevailed in these office workers (Table 2). The pain symptoms reported in our study were similar with the previous studies that neck and shoulder pain were commonly suggested to be work-related[24–26]. The participants in this study, on average, worked more than 45 hours per week and the average break time during work days was only ~35 minutes/day (Table 1). Working without adequate breaks had resulted in a significant correlation between pain symptoms and the break time during workday (p<0.05) (Table 3). An early study of upper extremity pain of computer users showed that the heightened job stress and the tendency to continue to work in a way to ensure high quality were related to pain intensity at work and the decreased function[4]. The induced neck/shoulder pain of the SOW in a fixed posture over long periods of time had also been observed in our study.

### Perfusion Characteristics in Pain-afflicted Office Workers

Wahlström mentioned Knardahl’s hypothesis that blood vessel–nociceptor interactions are of central importance in generating pain for work situations with cognitive tasks and low level muscle contractions. However, Knardahl’s hypothesis raises the need to rethink the methods and concepts used for studies of myalgia and musculoskeletal symptoms and pain associated with “visual display units” use[27]. Generally, the degree of blood perfusion in the cutaneous microvascular bed can provide a good indicator of peripheral vascular disease and can be indicative of the overall health of the vascular system[28]. Therefore, the intensity (mean microcirculatory blood flow, MMBF) and the amplitude (pulsatile microcirculatory blood flow, PMBF) of pulsatile flow of tissue perfusion are used as parameters in our study to assess the shoulder microvascular health.

The PP values become moderately correlated with shoulder pain level (p = 0.07), which was attributed by rapid reduction of MMBF values with increasing pain level (Fig 1). And the MMBF values of the high-pain group were significantly lower than the values of the low-pain group (p<0.05). These findings are consistent with previous studies that degenerative tendon pathology may stem from hypoxia[29], and a sedentary lifestyle has been proposed as a main reason for poor basal circulation of the tendon[30].

Cagnie’s study with non-invasive technique showed that 1 hour of combined workstation tasks resulted in decreased oxygen saturation and blood flow in all three parts of the trapezius muscle[31]. During 1 hour typing work, case group with pain symptom showed lower blood flux on average. Similar observation was shown in Fig 1, when pain level became higher from 1 to 10, the MMBF value reduced rapidly. The blood perfusion of subjects with high-pain level (~8) was low, while there were larger inter-individual variations among subjects with low-pain level (≤2). Further, the MMBF values of the high-pain group were significantly lower (p<0.05). That means, rapid reduction of MMBF values with increasing pain level had resulted in the perfusion characteristics of lower MMBF values in the high-pain group. Therefore, we suggested that ischemia (lower MMBF values) might exist in the shoulder of office workers with high-pain level. And we proposed that the perfusion pulsatility (PP value) of blood flow might present as an indication of impaired microcirculation.

In contrast with Cagnie’s study, some studies indicated a significant association between vasodilation in muscle and perceived pain [20,32]. Two subjects with lower pain-level (level<2) (Fig 1) also showed higher blood perfusion. In Roe’s study, a marked hyperemia, which was induced during the tracking task that model computer work, was observed after cessation of tracking[32]. Further, Strom indicated that pain in the active side correlated positively with blood flux in the pain-afflicted subjects during 90 min of office-work. These data showed that pain was associated with trapezius vasodilation but not with muscle activity[20]. Their measurements were invasive and involved real-time monitoring during office-work. In our study, the skin perfusion of shoulder was measured using non-invasive technique in resting condition during the break time of workday, which was similar to the measurement after cessation of tracking recorded in ROE’s study. Therefore, we suggested that hyperemia (higher MMBF values) might exist in the shoulder of office workers with low-pain level.

An early study of Larsson et al. had shown that subjects with trapezius myalgia had impaired muscle blood flow in the painful side during static contraction at different levels[33], compared to controls. Based on our results obtained from the new developed LDF technique, the perfusion characteristic (PP value) in the microvascular bed seems to be a potential physiological indicator to investigate the work-related shoulder pain. However, just as Jensen’s conclusion that the precise mechanisms involved in different microvascular beds during hypoxia are not well resolved[34]. More in-depth studies were still needed to verify the relationship between the perfusion characteristics and the cause of pain.

### Aging Effect on Microcirculation and Pain Symptoms

As mentioned above, microcirculatory blood flow of the most painful shoulders might be in a status of ischemia. Generally, peripheral microcirculation tends to degrade in the amount of blood flux by aging effect. Regarding aging effect, Brande et al concluded that the mean microcirculatory flux and the mean flow motion amplitude under resting conditions at the skin of the dorsum of the foot were significantly reduced in an old age group[35]. Thus, we further analyzed the aging effect on microcirculation. In consistence with their studies, the PMBF and MMBF values measured in our study were also significantly and negatively correlated with age (p<0.05) (Table 3). A major risk factor, age, is for the pathogenesis of vascular disease, and advancing age is associated with impaired arterial elasticity and endothelial function[36]. Advancing age with decreased cardiac output and arteriole compliance might lead to the reduction of resting microvascular blood flux. That means aging also have effects on the degraded microcirculation of office workers who suffered longer pain duration, but not necessarily lead to a higher pain-level of shoulder as shown in Table 3. Furthermore, from Brande’s study[35], there was a ~20% reduction in the blood flow from age of 25 to 70. If workers with MMBF>100 were excluded, there was a 54% reduction in the blood flow from age of 23 to 47 in the SOW. There was approximately a reduction of 1.7 AU per year for the office workers with sedentary lifestyle. Our result additionally implied that the impairment of microcirculation induced by long-term sedentary lifestyle may overtake that of the ageing effect.

Regarding the occupational factors, we found the duration of employment was also strongly associated with the pain duration, shorter break time during workday also strongly associated with pain symptoms (Table 3). These results may be a plausible connection with the long-term sedentary lifestyle of office workers. Similar results were found in a study of elderly female computer-users in EU countries, there was as many as 60% of those with self-reported neck symptoms of a certain duration and intensity, with trapezius myalgia (38%), tension neck syndrome (17%), and cervicalgia (17%) being the most frequent[37]. Further considering Crutchfield’s discussion that as vessels become diseased, they lose the ability to maintain this type of pulsatile flow[38], i.e. an indication of a lower PMBF value. Impaired microcirculation caused by the age effect, when coupled with sedentary lifestyle, are more likely to evoke ischemia pain which may persist a considerable period of time.

In summary, our study is to compare the objective (LDF measurement) and subjective (NMQ survey) indicators of shoulder health, the variations in the perfusion characteristics of the SOW may be explained as follows: (1) High-pain group with significantly lower MMBF values was postulated due to ischemia. (2) Impairment of shoulder perfusion due to aging effect may be worsened when combined with long-term sedentary lifestyle. (3) High MMBF values (>100) in the low-pain group was probably associated with hyperemia. (4) More robust data are needed to incorporate the current indicator, PP value, to evaluate the perfusion characteristics of the SOW.

Small sample size in this study was due to the requirements of pain symptoms and sedentary lifestyle for the participants. In addition, the limitations of the study include the single point measurement and the variation in the individual pain perception, which may lead to statistically non-significant (PP value). Thus, the results of this study should be interpreted with caution. Other factors which may affect the LDF measurements include BMI, drinking coffee, staying up late, and low surrounding temperature during measurement, etc. However, the non-invasive LDF technique used in this study is as convenient as measuring blood pressure. And the LDF technique, which successfully characterized the change of blood perfusion in our study, seems to provide a new direction in the investigation of work-related shoulder pain.

## Conclusions

In addition to questionnaires, the LDF technique could be a complementary and objective tool for the assessment of neck/shoulder health. In this study, lowering values of the PMBF were found for the sedentary office workers which suffered longer pain duration. And the MMBF of the high-pain group had significantly lower values. Although the general physiological parameters of most participants were within the normal range, impaired microcirculation caused by age effect, when coupled with sedentary lifestyle, are more likely to evoke ischemia pain. The LDF methodology also distinguished the perfusion pulsatility (PP value) with higher shoulder pain level, although this was not significant. However, insufficient blood perfusion at high-pain level might be used as a preventive indicator of ischemia pain. Further studies were required to investigate the underlying mechanisms of pain caused by sedentary lifestyle.

## Supporting Information

S1 Data Data of the study. (DOCX)
