# Supplementary material for: Correlations of Neck/Shoulder Perfusion Characteristics and Pain Symptoms of the Female Office Workers with Sedentary Lifestyle
Source: PLoS One. 2017 Jan 6;12(1):e0169318. doi: 10.1371/journal.pone.0169318 (PMC5217948; doi:10.1371/journal.pone.0169318)
Supplement: S1 Data — (DOCX) [file pone.0169318.s001.docx]

**Supporting Information**

**S1 Data of the study**

| subject number | age | BMI | shoulder pain level | Duration of shoulder pain | Effect of shoulder pain | MMBF | PMBF |
| --- | --- | --- | --- | --- | --- | --- | --- |
| 1 | 38 | 19 | 0 | 0 | 0 | 48.0 | 10.3 |
| 2 | 25 | 24 | 0 | 0 | 0 | 65.0 | 9.3 |
| 3 | 26 | 19 | 0 | 0 | 0 | 70.0 | 8.9 |
| 4 | 42 | 17 | 0 | 0 | 0 | 62.7 | 9.6 |
| 5 | 32 | 17 | 0 | 0 | 0 | 74.0 | 7.0 |
| 6 | 34 | 21 | 6 | 2 | 2 |  |  |
| 7 | 25 | 17 | 0 | 0 | 0 | 90.0 | 15.1 |
| 8 | 31 | 25 | 3 | 1 | 2 | 47.0 | 6.6 |
| 9 | 33 | 20 | 8 | 4 | 2 | 46.0 | 10.4 |
| 10 | 30 | 22 | 4 | 6 | 2 |  |  |
| 11 | 27 | 18 | 0 | 0 | 0 | 46.0 | 5.3 |
| 12 | 26 | 21 | 6 | 1 | 2 | 70.0 | 14.9 |
| 13 | 29 | 21 | 4 | 4 | 2 | 88.0 | 14.3 |
| 14 | 24 | 17 | 0 | 0 | 0 | 200.0 | 18.0 |
| 15 | 23 | 17 | 3 | 1 | 2 | 65.0 | 7.0 |
| 16 | 47 | 23 | 4 | 6 | 1 | 30.0 | 4.0 |
| 17 | 36 | 25 | 3 | 6 | 1 | 49.0 | 6.0 |
| 18 | 26 | 25 | 5 | 4 | 2 | 80.0 | 12.0 |
| 19 | 35 | 27 | 4 | 1 | 1 | 32.0 | 4.0 |
| 20 | 41 | 23 | 2 | 6 | 1 | 34.0 | 6.0 |
| 21 | 28 | 19 | 0 | 0 | 0 |  |  |
| 22 | 33 | 18 | 4 | 2 | 2 | 29.0 | 5.0 |
| 23 | 27 | 22 | 6 | 4 | 2 | 50.0 | 9.0 |
| 24 | 24 | 25 | 0 | 0 | 0 |  |  |
| 25 | 31 | 23 | 0 | 0 | 0 |  |  |
| 26 | 44 | 23 |  |  | 1 | 46.0 | 5.8 |
| 27 | 36 | 29 | 2 | 1 | 5 | 174.0 | 18.9 |
| 28 | 43 | 21 | 4 | 4 | 2 | 49.0 | 4.3 |
| 29 | 35 | 30 | 8 | 4 | 3 | 36.0 | 4.4 |
| 30 | 25 |  | 2 | 1 | 1 |  |  |
| 31 | 36 | 21 | 3 | 6 | 1 |  |  |
| 32 | 38 | 23 | 2 | 6 | 1 | 154 | 16.69 |
| 33 | 40 | 21 | 2 | 2 | 2 | 45 | 4.92 |
| 34 | 29 | 34 | 0 | 0 | 0 | 48 | 4.59 |
| 35 | 24 | 30 | 0 | 0 | 0 | 54 | 7.94 |
| 36 | 33 | 34 | 2 | 6 | 1 | 56 | 3.36 |
| 37 | 35 | 21 | 6 | 6 | 3 | 42 | 2.58 |
